# Balancing Innate Immunity and Inflammatory State via Modulation of Neutrophil Function: A Novel Strategy to Fight Sepsis

**DOI:** 10.1155/2015/187048

**Published:** 2015-12-21

**Authors:** Haoshu Fang, Wei Jiang, Jin Cheng, Yan Lu, Anding Liu, Lixin Kan, Uta Dahmen

**Affiliations:** ^1^Department of Pathophysiology, Anhui Medical University, Hefei 230032, China; ^2^Experimental Medicine Center, Tongji Hospital, Tongji Medical College, Huazhong University of Science and Technology, Wuhan 430030, China; ^3^Department of Neurology, Feinberg School of Medicine, Northwestern University, Chicago, IL 60611, USA; ^4^Experimental Transplantation Surgery, Department of General, Visceral and Vascular Surgery, Jena University Hospital, 07747 Jena, Germany

## Abstract

Sepsis and SIRS (systemic inflammatory response syndrome) belong to a severe disease complex characterized by infection and/or a whole-body inflammatory state. There is a growing body of evidence that neutrophils are actively involved in sepsis and are responsible for both release of cytokines and phagocytosis of pathogens. The neutrophil level is mainly regulated by G-CSF, a cytokine and drug, which is widely used in the septic patient with neutropenia. This review will briefly summarize the role of neutrophils and the therapeutic effect of G-CSF in sepsis. We further suggest that targeting neutrophil function to modulate the balance between innate immunity and inflammatory injury could be a worthwhile therapeutic strategy for sepsis.

## 1. Introduction

SIRS (systemic inflammatory response syndrome) and sepsis are two different entities of the same disease complex both leading to multiorgan dysfunction and eventually death of the patient.

SIRS is defined as an overwhelming systemic inflammation without infectious component. In contrast, sepsis is a potential fatal medical condition that is characterized by a severe systemic infection accompanied by a dysregulated systemic inflammation [1]. Experimentally, SIRS is often induced by injection of LPS, whereas the frequently used model of sepsis is based on the intra-abdominal inoculation of fecal suspension.

The causes for SIRS and sepsis could be manifold. SIRS can develop as sterile complication of severe trauma, extensive burns, shock, or severe local inflammation such as pancreatitis. The concomitant inflammatory response to the increasing endotoxin levels may result in a vicious cycle leading to SIRS. Sepsis can result from any local or systemic infection and is frequently associated with increased blood levels of endotoxin.

Severe sepsis is a major cause of death in the intensive care unit (ICU) of hospitals and affects millions of people around the world each year. Despite an overwhelming increase in our knowledge regarding the pathogenesis of sepsis and the subsequent advances in clinical care, sepsis still accounts for an unacceptable high mortality ranging from 25 to 30% [2].

## 2. Neutrophils Are the Major Cell Type Involved in SIRS and Sepsis

### 2.1. Neutrophil Is the Primary Line of Defense against Infection

The neutrophils are the major cell type of the innate immune system, which acts as primary line of defense against invading microbial pathogens [3]. Neutrophils are terminally differentiated hemopoietic cells with a short life span, which respond to infection by migrating from the bloodstream into the infectious site [3].

Efficiency of bacterial elimination is dependent on the rapid recruitment of neutrophils from the circulation, which is promoted by the release of chemotactic agents [4]. Once at the site of infection, neutrophils capture the microbe into a phagosome, which then fuses within transcellular granules forming a phagolysosome. In the phagolysosome, the microbe is destroyed by a combination of oxidative (reactive oxygen species; ROS) and nonoxidative (enzymes, proteases, and antimicrobial peptides) mechanisms [3].

### 2.2. The Activation of Neutrophils Induces an Overt Inflammatory Response and Causes Tissue Damage

Although neutrophils are important for pathogen clearance, the activation of neutrophils also causes an overt inflammatory response and tissue injury. The migration of neutrophils could potentially extend neutrophil-endothelial cell interactions and enhance vascular damage [4]. Local secretion of cytokines by the neutrophils might change the nonthrombogenic properties of endothelial cells to a procoagulant state with the initiation of disseminated intravascular coagulation (DIC) and induce the production of nitric oxide in both endothelial and smooth muscle cells [4]. The inducible nitric oxide (iNOs) is mainly released by neutrophils and has received considerable attention as a mediator of the tissue response to sepsis [5]. The key function of iNOs is to induce the synthesis of nitric oxide (NO), which leads to vasodilation, cytotoxicity, and inflammation [6].

## 3. G-CSF Mobilizes Neutrophils and Enhances the Innate Immunity

### 3.1. Mobilization of Neutrophils Is Induced by Granulocyte Colony-Stimulating Factor (G-CSF)

G-CSF is the principal granulopoietic growth factor regulating the maturation, proliferation, and differentiation of neutrophil precursors and has been used in patients with neutropenia [7]. It enhances maturation of neutrophil functions such as chemotaxis, phagocytes, and bactericidal clearance. It suppresses production of tumor necrosis factor-*α* (TNF-*α*) and promotes the release of IL-1ra and soluble TNF receptor (sTNFr) [8]. It is formed at the site of infection or inflammation but exerts its primary action at a remote organ, that is, bone marrow [8]. G-CSF is able to stimulate the proliferation of neutrophils and plays an important role in modulating the release of inflammatory mediators in acute inflammation.

### 3.2. G-CSF Is Modulating the Innate Immune Response

Several studies have documented the neutrophil-activating effect of G-CSF [9], indicating that it should be considered a potent activator of mature circulating neutrophils. It is capable of priming the respiratory burst, inducing the release of secretory vesicles, and modulating the expression of surface adhesion molecules. The polymorphonuclear surface antigen CD11b/CD18 expression and the plasma elastase-*α*1AT complex levels are increased following G-CSF administration.

G-CSF induced mobilization of CD34^+^ cells and improved survival of patients with acute-on-chronic liver failure (ACLF) [10]. This effect was attributed to the increased innate immune state, which potentially contributed to prevention from sepsis and multiorgan failure, and improved survival in the study group [11]. A recent experimental study has shown that the use of G-CSF recruited bone marrow-derived macrophages into the liver, which, on engraftment in the liver, did help in reducing the hepatic fibrosis and supported hepatic regeneration.

## 4. G-CSF Is Used for Treatment of Septic Patients

### 4.1. G-CSF Treatment Increased Immune Response of the Patients

According to a large number of clinical trials, G-CSF can decrease the incidence of infections and strengthen host defense in patients. G-CSF administration increased concentrations of IL-1ra, soluble TNF receptors (sTNFr), and IL-10 and reduced TNF-*α*, IFN-*γ*, and GM-CSF in healthy volunteers [12]. Application of a single dose of G-CSF resulted in the upregulation of neutrophils. These newly formed neutrophils were well equipped against bacterial infections in terms of Fcy RI expression, Fcy RI-dependent antibody-dependent cellular cytotoxicity, and strong-surface CD14 expression. In contrast the biological significance of the decreased surface-expression of Fcy RI11 and the high intracellular LAP content still needs to be elucidated.

### 4.2. G-CSF Has Beneficial Effects in Experimental Models of Sepsis

G-CSF has been used successfully in the past to prevent and to treat experimentally induced sepsis, for example, using the cecal ligation and perforation (CLP) model in rats and mice [13, 14]. It has been reported that the combination of G-CSF application and antibiotic prophylaxis was the most efficacious treatment in rats with polymicrobial peritonitis [15]. Prophylaxis with G-CSF increased the survival rate, decreased the bacterial load, and promoted the production of inflammatory cytokines directly, as demonstrated in experiments [16].

The protective effects were at least partially related to increased neutrophil function, as studies proved that similar phenomena were observed by increasing the neutrophil secretory proteins. Singer observed in a model of iNOs knock-out mice that the iNOs derived NO is a determinant of the proinflammatory phenotype acquired by the hepatic microvasculature during sepsis [5]. Luo et al. found that the neutrophil extracellular trap had a proinflammatory role in abdominal sepsis and regulated the pulmonary infiltration of neutrophils and tissue injury [17].

The results might provide insights and guidance for the application of G-CSF and antibiotic in the perioperative treatment of patients who are susceptible to infection following intra-abdominal surgery.

### 4.3. G-CSF Treatment of Patients with Sepsis Leads to Controversial Results

**Figure 1:**
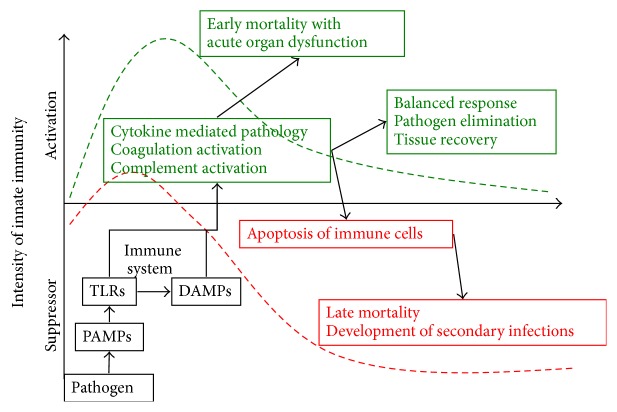
The development of sepsis has two phases, that is, activating and suppressing phase. The activation of the innate immune system can lead to a balanced response that can trigger the elimination of invading pathogens and the recovery of tissue but can also lead to an unbalanced response that can induce hyperinflammation or immune suppression.

It has become well accepted that sepsis is composed of two, often concomitant, phases (Figure 1): an immune-activated phase and an immune-suppressed phase [1]. Identification of two distinct phases of sepsis calls for a stage dependent therapy. However, it remains difficult since the two stages can be hardly distinguished in experimental models but much less in patient with sepsis.

Therefore, treatment strategies targeting only one phase may fail and cause mortality.

G-CSF is frequently and successfully used in patients with severe neutropenia, who are at risk of sepsis [18]. However, when used in clinical trials to treat ongoing severe sepsis or when given as prophylactic treatment, G-CSF did not result in a clear benefit (Table 1). It is reported that perioperative G-CSF administration was effective in upregulating immune function in patients subjected to major surgery. G-CSF administration resulted in increased levels of natural circulating antagonists of TNF-*α* and IL-1, that is, TNF-R p55/p75 and IL-1ra in patients, thus increasing the threshold of triggering the inflammatory reaction. Perioperative prophylaxis with G-CSF in high-risk colorectal cancer patients resulted in improved recovery [19]. In acute-on-chronic liver failure (ACLF) patients, treatment of G-CSF significantly decreases the risk of sepsis [10]. Additionally, G-CSF is used in the treatment of neonates and adults with infection [20].

Although G-CSF administration is associated with a longer duration of survival in patients with severe sepsis, a meta-analysis from Bo et al. indicated that G-CSF therapy did not significantly reduce the overall mortality at 14 days or 28 days or in-hospital mortality in patients with sepsis [21]. Nelson et al. reported that G-CSF treatment did effectively increase neutrophil levels but did not affect the mortality rate of the patient with community-acquired pneumonia [22]. A multicenter clinical trial of G-CSF had shown similar results that the G-CSF treatment did not substantially reduce mortality and complication rate in patients with pneumonia and severe sepsis [23]. The clinical trials in nonneutropenic septic patients indicated that the use of G-CSF decreased the risk of sepsis in nosocomial pneumonia patients, although the effect did not reach statistical significance [24]. Similarly, a clinical observation from melioidosis patients indicated that G-CSF treatment was associated with longer survival duration but was not associated with an overall survival benefit [25]. Another study from the same group showed that the G-CSF treatment did not improve the outcome in patients with septic shock, [26]. Altogether, the G-CSF administration in infectious disease was not associated with a clear therapeutic benefit (Table 1).

## 5. The Therapeutic Effect of G-CSF Might Be Enhanced by Reducing the Inflammatory Response

### 5.1. G-CSF Treatment Induces LPS Sensitization

Our own results demonstrated that G-CSF pretreatment was not only mobilizing neutrophils but also inducing LPS sensitization to inflammatory response [27]. The inflammatory response contributed to bacterial clearance but did become deleterious if circulating LPS was abundant. Therefore, both components of the disease—the state of innate immunity and the inflammatory response—have to be addressed appropriately to maximize the therapeutic efficiency of GCSF in sepsis.

According to the sepsis treatment guidelines [28–30], therapy consists of elimination of the septic focus by early antibiotic treatment (“hit early, hit hard”) as well as supportive therapy consisting of volume resuscitation and maintenance of organ function. Antibiotic treatment is very efficient to reduce the bacterial load. However, the antimicrobial, cytolytic properties of antibiotics may induce the release of LPS from the outer membrane of Gram-negative bacteria [31]. Antibiosis does not address the effect of high circulating endotoxin levels. Current guidelines do not call for reducing the overwhelming inflammatory response to circulating free endotoxin. New evidence indicated that the controversial clinical observations might be related to the G-CSF modulated inflammatory response.

### 5.2. Strategies to Decrease the LPS Induced Inflammatory Response

A number of strategies exist to reduce circulating LPS levels or to minimize the response to LPS [32]. Elimination of LPS using polymyxin columns is troublesome because of the unproven benefit, significant costs, and potential risks [33]. Neutralization of LPS using antibodies reduced the LPS induced inflammatory response [32]. Currently, vaccination strategies to increase the elimination of pathogens are under investigation [34]. None of these antiendotoxin strategies reached wide clinical acceptance.

Minimizing the response to LPS was explored by blocking different pathways. Blockade of the interaction between LPS and TLR4 signal pathway decreased TNF-*α* levels in LPS induced SIRS model and* E. coli* induced sepsis model [35]. The intracellular signal transduction cascade can be blocked to prevent the excessive induction of proinflammatory cytokines. However, clinical trials using TNF-*α* antibodies were not promising [36].

Recently TLR4 blockade gained increasing attention [37]. The TLR4 signaling pathway leading to LPS-mediated NF-kappa B activation constitutes an important therapeutic target for sepsis therapy. Various molecules are involved in regulating TLR4-expression on the cell membrane and act as new adjuvant therapies that are able to weaken the deleterious effects of exaggerated host response to infection [38].

Eritoran tetrasodium is a nonpathogenic endotoxin analog that antagonizes inflammatory signaling via the immune receptor TLR4 [39]. Therefore, eritoran tetrasodium (E5564) was investigated as promising molecular candidate to treat sepsis [40]. Christ et al. proposed that E5531, a slightly different analogue, would antagonize LPS activity at its cell-surface receptor leading to inhibition of transmembrane signal transduction [41]. E5531 protected mice from lethal doses of LPS and from viable* E. coli* infections in combination with antibiotics. However, E5531 did not affect bacterial counts. In contrast, additional administration of antibiotic dramatically decreased blood bacterial counts, but plasma endotoxin levels were concomitantly increased in these animals.

### 5.3. LBP Blockade Is a Novel Strategy to Reduce LPS Induced Inflammatory Response

Our previous study indicated that G-CSF pretreatment induced upregulation of LPS binding protein (LBP) [27]. LPS binding protein (LBP) is an acute phase plasma protein with a molecular weight of 60 KD that can be detected in the acute phase serum of different species such as mice, rabbits, and human [42]. The serum LBP binds to the lipid A component of bacterial endotoxin and facilitates its transfer to the CD14 antigen, which is needed for triggering the inflammatory response via TLR4-NF*κ*B signaling pathway [43]. Martin et al. reported that the binding between LBP and LPS increased the bioactivity of LPS by 100 to 1000 times [44], playing an important role in triggering the inflammatory response in adult respiratory distress syndrome patients and rabbit, respectively [45]. However, upregulation of LBP, in turn, did augment the inflammatory response [46, 47]. It has been shown in different models that upregulation of LBP prior to an LPS challenge potentiates the inflammatory response which may largely contribute to LPS toxicity in sepsis [46].

Minimizing the inflammatory response to LPS can be achieved by interfering with the interaction of LPS and LBP. The inflammatory response to endotoxin can be decreased by reducing circulating endotoxin levels or by reducing the response of the organism to circulating endotoxin. Experimental strategies include using LBP inhibitory peptide [35], or LPS analogues [48], or using LBP deficient mice [47, 49]. Knapp et al. reported [50] that LBP(−/−) mice were associated with diminished early tumor necrosis factor alpha, interleukin-6, cytokine-induced neutrophil chemoattractant, and macrophage inflammatory protein production and attenuated recruitment of polymorphonuclear leukocytes to the site of infection, indicating that acute inflammation was promoted by LBP. However, LBP(−/−) mice were highly susceptible to* E. coli* peritonitis, as indicated by increased mortality, earlier bacterial dissemination to the blood, impaired bacterial clearance in the peritoneal cavity, and more severe remote organ damage.

Le Roy et al. demonstrated that neutralization of LBP accomplished by blocking either the binding of LPS to LBP or the binding of LPS/LBP complexes to CD14 protected the host from LPS induced toxicity [51]. Araña et al. showed that application of the LBP inhibitory peptides blocked the LBP-LPS interaction efficiently and prevented death of animals in an endotoxin shock model by suppressing the TNF-*α* response to an LPS challenge [35]. This was confirmed in another publication using CLP-19, a synthetic peptide derived from* Limulus* (anti-LPS factor) [52].

## 6. Outcome of Sepsis Was Improved by Modulating Neutrophil Function and Inflammatory Response

The role of G-CSF pretreatment and subsequent LBP upregulation was investigated in a SIRS model and a sepsis model. Interestingly, In the SIRS model, G-CSF pretreatment enhanced and accelerated the uptake of LPS by the liver. Subsequently, G-CSF pretreatment caused an overwhelming inflammatory response to LPS leading to the death of all animals in response to an otherwise sublethal dose of LPS. This response could at least be partially attributed to the upregulation of LBP prior to the LPS challenge, as blocking of LBP using an inhibitory peptide abrogated the effects of G-CSF pretreatment [27].

**Figure 2:**
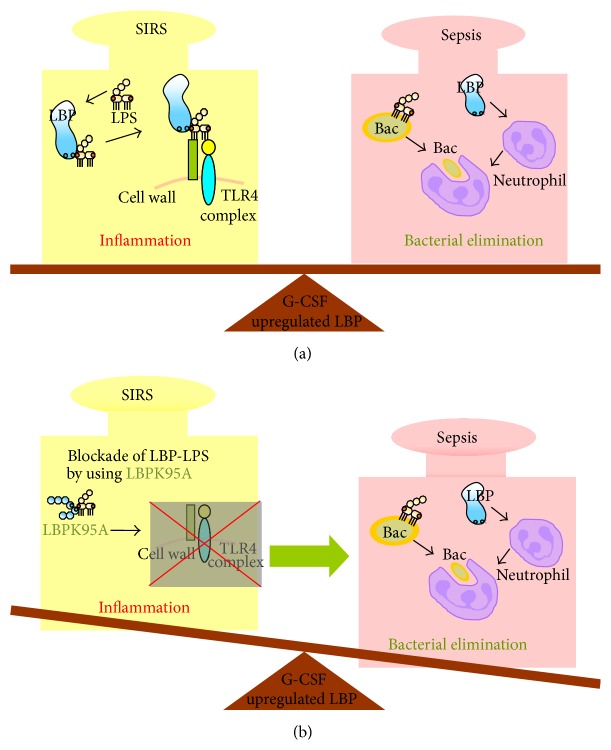
Balanced modulation of LBP-effect by LBP blockade and G-CSF pretreatment in sepsis. (a) on one hand, LBP promotes bacterial clearance, but on the other hand, it contributes to the sensitization of inflammatory response via activation of NF-*κ*B signaling pathway. (b) Interfering with the LBP-mediated inflammatory response by LBP blockade reduces the inflammatory injury and improves outcome after septic insult.

In contrast, in the sepsis model, G-CSF pretreatment was associated with an increased survival rate when compared with an untreated control group [53]. This was paralleled by a reduced inflammatory response. Of note, this response could also be at least partially attributed to the upregulation of LBP prior to the septic insult, as blocking of LBP using an inhibitory peptide abrogated the effect of G-CSF pretreatment. Moreover, LBP upregulation during the infection has seemingly a dual function. Yang et al. found that LBP deficient mice showed delayed neutrophil influx in case of a peritoneal infection [54]. This led to the idea that LBP might have a dual role: augmenting the inflammatory response to bacterial toxin such as LPS and contributing to bacterial elimination via the associated and enhanced neutrophil infiltration (Figure 2). These two functions might help to explain that either LBP blockade or G-CSF treatment alone was not useful in the therapy of patients with severe sepsis.

## 7. Conclusion

The seeming contradictory results above support the idea that augmenting the neutrophil response via G-CSF treatment is a two-edged sword. Elimination of bacteria by increasing the innate immunity seemed improved via the mobilization of neutrophils, whereas LPS response was augmented via upregulation of LBP [47]. Therefore, these evidences suggest that the therapeutic strategies by combining the increased bacterial elimination via improving the innate immunity and decreasing the inflammatory injury could be a worthwhile therapeutic strategy for sepsis. Further G-CSF-based therapeutic strategies should be designed to potentially combine with effects of increasing bacterial elimination via mobilization of neutrophil and decreasing the inflammatory response by blocking the LPS response.

## Figures and Tables

**Table 1:** Clinical studies investigating effect of G-CSF treatment on infectious disease.

Study	Design	Treatment groups	Disease	G-CSF treatment schedule	Conclusion	Effect of G-CSF
Garg et al., 2012 [10]	Effect of G-CSF on survival rate in patients with liver failure	Placebo G-CSF	Acute-on-chronic liver disease	5 *μ*g/kg	G-CSF decreased risk of sepsis significantly	Positive

Stephens et al. [26]	Effect of G-CSF on patients with septic shock	Placebo G-CSF	Septic shock	263 *μ*g/day, 3 days	G-CSF did not improve outcome in patients with septic shock	Indifferent

Cheng et al., 2007 [25]	Effect of G-CSF on severe septic patients with melioidosis infection	Placebo G-CSF	Melioidosis	263 *μ*g/day, 3 days	G-CSF was associated with a longer duration of survival but was not associated with a higher survival rate	Indifferent

Hartmann et al., 2005 [24]	Effect of G-CSF on pneumonia patients	Placebo G-CSF	Nosocomial pneumonia	300–480 *μ*g/day for 7 days	G-CSF decreased risk of sepsis but did reach significance	Indifferent

Hartung et al., 2003 [55]	Perioperative treatment of G-CSF before abdominal surgery	Placebo G-CSF	Mixture, operation	5 *μ*g/kg for 3 times or continuous administration of 5 *μ*g/kg for 5 days after an initial bolus of 5 *μ*g/kg	G-CSF decreased the postoperative infection	Positive

Root et al., 2003 [23]	Effect of G-CSF on pneumonia and sepsis patients	Placebo G-CSF	Pneumonia and severe sepsis	300 *μ*g/day for 5 days	G-CSF was not efficacious in reducing mortality and complications from infection	Indifferent

Tanaka et al., 2001 [56]	Lung injury was investigated after G-CSF treatment	Placebo G-CSF	Sepsis	2 *μ*g/kg for 5 days	G-CSF attenuated inflammatory response	Positive

Ishikawa et al., 2000 [8]	G-CSF with relative neutropenia septic patients	Absolute neutrophil count: high, moderate, and low	Mixture	2 *μ*g/kg for 5 days	G-CSF was effective in septic patient with a percentage of immature neutrophils, but less effect with high percentage of immature neutrophils and bone marrow was depressed.	Positive

Nelson et al., 1998 [22]	Effect of G-CSF on pneumonia patients	Placebo G-CSF	Community-acquired pneumonia	300 *μ*g/day for 10 days	G-CSF did increase neutrophil levels but did not affect the mortality of patient with pneumonia	Indifferent
